# Repression of GH signaling: One extended life to live!

**DOI:** 10.18632/aging.100608

**Published:** 2013-10-17

**Authors:** Darlene E. Berryman, Edward O. List, John J. Kopchick

**Affiliations:** ^1^ Edison Biotechnology Institute, Ohio University, Athens, OH 45701, USA; ^2^ Department of Biomedical Sciences, Heritage College of Osteopathic Medicine, Ohio University, Athens, OH 45701, USA; ^3^ School of Applied Health Sciences and Wellness, College of Health Sciences and Professions, Ohio University, Athens, OH 45701, USA

We have generated two independent dwarf mouse lines with decreased GH action; however, only one has extended longevity. Why?

Decreased signaling through the GH/IGF-1 axis in vertebrates, or comparable pathways in invertebrates, has repeatedly been shown to extend lifespan. A prominent example is mice with a disruption in the GH receptor gene (GHR−/−) generated in our laboratory two decades ago [1]. GHR−/− mice are completely resistance to GH action, which causes a reproducible extension of lifespan regardless of the genetic strain of mice [1, 2] and is officially recognized as the longest-lived laboratory mouse (http://methuselahfoundation.org/).

In 1991, our laboratory first described another dwarf mouse line that expresses a growth hormone receptor antagonist (GHA) transgene [3]. The expressed transgene is a mutated bovine GH gene in which the codon for the smaller glycine amino acid at position 119 is replaced with a larger amino acid, which causes steric hindrance when it interacts with the GHR resulting in a classical receptor antagonist [4]. This work ultimately led to the discovery of a pharmaceutical agent, pegvisomant, for the treatment of acromegaly. However, besides providing the basic information for development of this therapeutic, these GHA transgenic mice also provide a novel mouse strain to assess the outcome of a reduction in GH action on health and aging.

**Figure 1:**
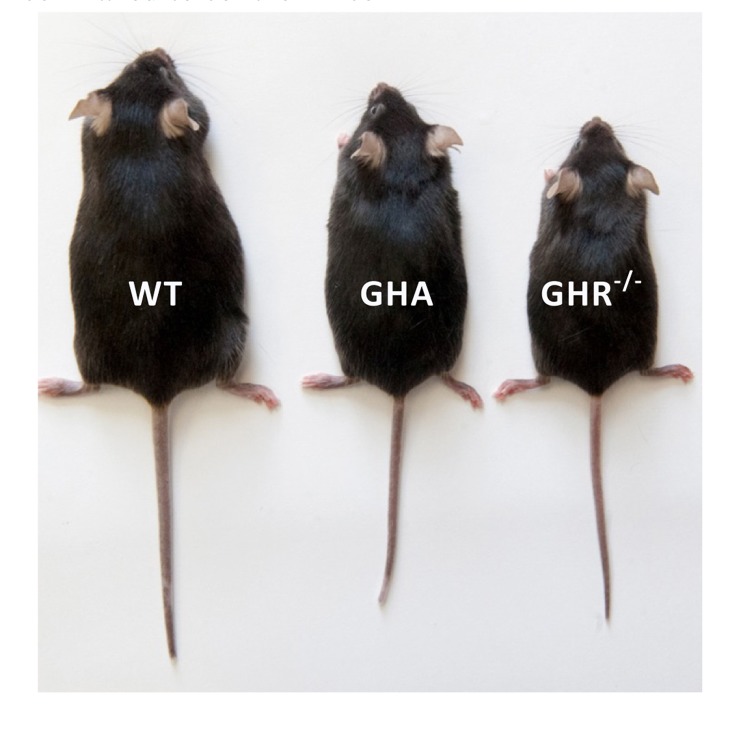
Phenotypic comparison between GHA mice and long-lived GHR−/− mice as compared to control mice (WT).

Noteworthy is the fact that GHA mice do not experience significantly longer lifespans as do other mouse lines with a reduction in the GH/IGF-1 axis, such as the aforementioned GHR−/− mice [2]. As a result, GHA mice have not been as extensively studied. Regardless, comparing the phenotype of GHA mice with other long-lived lines, such as GHR−/− mice, should reveal the most important traits caused by reduced GH action that are responsible for lifespan extension. A summary comparing the phenotypes of GHR−/− and GHA mice is provided in Figure 1. An important distinction between GHA mice and GHR−/− mice is that the GHA does not completely inhibit GH signaling, while inhibition of GH signaling in GHR−/− mice is complete. Thus, we have generated two dwarf mice each with either low or no GH induced intra-cellular signaling (and each with low levels of IGF-1) yet only one has extended longevity.

Again, what molecular mechanisms account for this difference in lifespan between these two dwarf lines? GHA mice generally have a phenotype intermediate between that of control and GHR−/− mice, especially as it relates to size, readouts of the GH/IGF-1 axis and measures of glucose homeostasis. For example, GHA mice are dwarf, but not as dramatic as seen in GHR−/− mice [2]. As compared to controls, circulating IGF-1 are reduced in both lines but by only ~25-40% in GHA mice as opposed to >80% in GHR−/− mice [2]. While GHR−/− mice are extraordinarily insulin sensitive, glucose homeostasis is moderately improved in young GHA mice with low to normal plasma levels of glucose and insulin [2, 5]. However, insulin levels deteriorate with advancing age in male GHA mice [2, 6]. Perhaps the more marginal decreases in IGF-1 or the lack of dramatic alterations in glucose metabolism are sufficient in GHA mice to curb significant gains in longevity.

Interestingly, while dwarf throughout life, the body weight of male GHA mice gradually catches up to that of control mice with advancing age [2, 6]. The increase in body weight in later adult life is not due to increases in body length or lean tissue; thus, it is not due to catch up growth. Rather, the increase in body weight is due to marked increases in adipose tissue [6]. Where do GHA mice deposit their adipose tissue and could that be relevant to longevity? Like GHR−/− mice, GHA mice display dramatic increases in the subcutaneous fat depot [5, 7]. However, unlike GHR−/− mice, intra-abdominal fat pads (including visceral depots) become enlarged with advancing age in GHA mice, which may contribute to their deterioration in glucose homeostasis over time [6]. Despite many similarities in the adipokine profiles of GHR−/− and GHA mice (elevated leptin, adiponectin and resistin), only GHA mice experience a very dramatic increase in leptin levels with age that coincides with their progressive obesity [5-8]. With their severe obesity at older ages, it is curious that GHA mice do not live any shorter than littermate controls. Thus, repression of GH signaling via expression of the antagonist appears to confer some protection from complications that are commonly associated with obesity.

How do GHA mice compare to GHR−/− in tumor incidence, cardiovascular function, lipid metabolism, cognition, and other measures of health? More studies are needed. Nevertheless, GHA mice are valuable tools as they defy the common pathologies that accompany excess fat mass, and they offer an exception to the dogma that a decrease in GH action increases lifespan. But more importantly, GHA mice are likely a more clinically relevant mouse line to study than GHR−/− mice; after all, repression of GH action is achievable through pharmacological intervention with the use of a GHA (pegvisomant) whereas total repression of GH action, as in GHR−/− mice, is not nor would it be clinically desirable. Therefore, a better evaluation of the GHA phenotype, disease status, and metabolic state in both sexes and across lifespan is warranted.
